# Mapping Research Trends on the Implications of Telemedicine for Healthcare Professionals: A Comprehensive Bibliometric Analysis

**DOI:** 10.3390/healthcare13101149

**Published:** 2025-05-15

**Authors:** Chiara Bernuzzi, Maria Adele Piccardo, Chiara Guglielmetti

**Affiliations:** Department of Economics, Management and Quantitative Methods (DEMM), University of Milan, Via Conservatorio, 7, 20122 Milan, Italy; mariaadele.piccardo@unimi.it (M.A.P.); chiara.guglielmetti@unimi.it (C.G.)

**Keywords:** telemedicine, healthcare professionals, wellbeing, work outcomes, bibliometric analysis

## Abstract

**Background/Objectives:** The digital transformation in healthcare is reshaping care delivery by enhancing patient care and flexibility. However, it also poses potential challenges to healthcare professionals’ wellbeing and work practices. To date, research on the implications of telemedicine for healthcare professionals remains limited and inconclusive. This study aims to provide a comprehensive overview of this research field using a quantitative, bibliometric approach. **Methods:** Articles were systematically selected from Web of Science and Scopus databases, focusing on empirical, peer-reviewed articles written in English, involving healthcare professionals and focusing on telemedicine. **Results:** The dataset consists of 160 papers. The analysis reveals a significant increase in publications starting from 2012, with a notable surge in 2020, reflecting the impact of the COVID-19 pandemic. The University of New Mexico and the Cleveland Clinic Foundation, both in the United States, were identified as the institutions with the highest number of published articles. Most studies were published in clinical-focused journals (e.g., Journal of Medical Internet Research and BMC Health Services Research), emphasizing the field’s dominant orientation. The intellectual structure reveals that wellbeing, work practices, and communications between patients and professionals are central themes. **Conclusions:** This bibliometric analysis provides scholars with a clearer understanding of the intellectual structure of research on the implications of telemedicine for healthcare professionals, addressing key gaps left by previous reviews. While telemedicine offers numerous advantages, such as enhanced access to care and greater flexibility, it also raises challenges related to healthcare professionals’ wellbeing, work practices, and communication with patients. Both contextual factors (e.g., digital skills training) and individual characteristics (e.g., attitudes toward telemedicine) play a significant role in shaping healthcare professionals’ experiences with telemedicine. By identifying influential contributors and thematic patterns, this study offers a foundation for future research and informs the development of targeted interventions to sustain healthcare professionals in digitally mediated care environments.

## 1. Introduction

In recent years, telemedicine has become a key component of healthcare delivery by offering innovative solutions for patient care, especially in remote and underserved areas [1]. Telemedicine involves the use of digital communication and information technologies by medical professionals to deliver essential healthcare services, enabling the exchange of accurate information necessary for diagnosis, treatment, and prevention, regardless of time and location constraints [2]. This term encompasses a wide array of applications, such as remote consultations, distance health monitoring, and telerehabilitation [3]. It has the potential to improve access to care, streamline services, and enhance patient outcomes [4]. The relevance of telemedicine has surged particularly in the context of the COVID-19 pandemic, during which it enabled the provision of care at a distance, avoiding physical contact between patients and healthcare providers [5,6]. Telemedicine is now recognized not only for its capacity to enhance access to care, but also for its transformative effects on healthcare practice. To date, much of the research in this field has focused on technological advancements and on the effects of telemedicine on patient care [7]. However, healthcare professionals (i.e., individuals in the medical field, including physicians, nurses, and allied healthcare professionals responsible for delivering healthcare services to patients; HCPs [8]) are the ones implementing and managing these technology-driven services. This suggests the need to understand how telemedicine impacts HCPs, to ensure its successful and sustainable integration into healthcare systems.

Previous research has shown that the introduction of new technologies in the workplace often leads to changes and challenges [9], which can affect workers’ wellbeing. In the healthcare sector, digitalization represents both an opportunity and a burden [10]. On the one hand, it offers benefits such as greater flexibility [11], increased autonomy, and improved patient care [7]; on the other hand, it may exacerbate issues such as higher workload [12] and the erosion of traditional patient–professional relationships [11]. Telemedicine, therefore, is a double-edged sword: while it can optimize healthcare delivery, it also presents significant challenges for HCPs [13]. Despite a growing body of research examining HCPs’ acceptance of telemedicine tools [14,15], studies specifically addressing the implications of telemedicine on HCPs’ wellbeing remain scarce and fragmented, often producing mixed or context-specific results [16,17]. This represents a critical gap, as HCPs play a fundamental role in ensuring high-quality, safe, and sustainable healthcare. Indeed, prior studies have shown that distress and fatigue among physicians are associated with an increased risk of medical errors [18]. Although scholarly interest in telemedicine has intensified—particularly in the wake of the COVID-19 pandemic—most research has primarily focused on technological advancements, clinical applications, and patient outcomes, with considerably less attention given to HCPs’ perspectives. Even when reviews are available, they tend to concentrate on specific types of telemedicine services (e.g., video consultations [19]) or on HCPs’ attitudes and digital competencies [14,20,21,22], while largely overlooking the implications for wellbeing and work practices. To our knowledge, no previous study has employed bibliometric analysis to systematically map the scientific literature on the implications of telemedicine for HCPs, identify influential contributors, and uncover thematic patterns and knowledge gaps. Such an approach is essential not only to conceptually organizing the field, but also to highlighting underexplored areas and guiding future research directions.

To address these gaps, this study presents a bibliometric analysis aimed at providing a structured overview of the scientific literature on the implications of telemedicine for HCPs. The objectives of this analysis are threefold: (1) to map the evolution and distribution of scholarly production in this area, identifying key contributors (authors, institutions, countries, and journals); (2) to explore the conceptual structure of this field by identifying major research themes and emerging topics related to HCPs’ experience with telemedicine; and (3) to highlight underexplored areas and provide suggestions for future research directions that could better support HCPs in digitally mediated care environments.

In line with these objectives, the following research questions guide this analysis:

(RQ1) Which journal(s), institution(s), and country(s) have the most publications in this research field?

(RQ2) What are the most researched themes and emerging topics related to HCPs’ experiences with telemedicine?

(RQ3) What are the relevant future directions for research in this field?

Through this comprehensive approach, we aim to provide a foundation for a more nuanced understanding of the challenges posed by telemedicine adoption among HCPs.

## 2. Materials and Methods

Bibliometric analysis is widely recognized as a robust and systematic tool for evaluating the scientific output of a research field [23]. Unlike other review methods, such as systematic or scoping reviews, bibliometric analysis adopts a macro-level perspective that enables researchers to trace the evolution of a field’s intellectual, social, and conceptual structures over time [23,24]. By grounding the review process in the statistical measurement of scientific production, scholars, and activity, this method ensures a systematic, transparent, and reproducible approach to knowledge mapping [25].

Crucially, bibliometric analysis relies on objective and replicable procedures, thereby minimizing interpretive bias and enhancing methodological rigor compared to more narrative or qualitative approaches [23,26]. In a moment of rapid growth and transformation in telemedicine, this approach provides strategic insights that traditional literature reviews may not fully capture. As telemedicine continues to reshape healthcare delivery, bibliometric methods offer a structured and data-driven overview of research trends and knowledge diffusion patterns, supporting scholars in navigating the complexity of this evolving field.

By offering a comprehensive and structured perspective, bibliometric analysis offers a solid foundation for the development of innovative research directions and the advancement of scholarly discussion [27].

The following sections detail the data collection and analysis processes, in accordance with the methodological guidelines proposed by Aria and Cuccurullo [25] and the guidelines for reporting bibliometric reviews in biomedical literature (BIBLIO) [28].

### 2.1. Data Collection

Documents were retrieved from two primary databases: Web of Science (WoS) and Scopus. These databases were selected for their comprehensive coverage of journals and articles with detailed metadata [29]. Regarding WoS, only articles indexed in the Science Citation Index Expanded (SCI Expanded), the Social Science Citation Index (SSCI) and the Emerging Sources Citation Index (ESCI) were considered. To capture a wide range of studies focusing on HCPs’ perspectives regarding the adoption of telemedicine tools, a search strategy was developed using the following keywords: physician* OR doctor* OR clinician* OR “healthcare manager*” OR “healthcare professional*” OR “healthcare worker*” OR “healthcare personnel” OR “health personnel” OR “health manager*” OR “health professional*” OR “health worker*” OR “medical staff” OR professional* OR nurse* AND telemedicine OR telehealth OR ehealth OR telemonitoring OR telecare AND wellbeing OR “professional wellbeing” OR “work-related stress” OR “job stress” OR “occupational stress” OR “work environment” OR workplace. The keywords were carefully selected to balance inclusivity and specificity, aiming to capture relevant studies while minimizing irrelevant results. In Scopus, the search focused on titles, abstracts, and keywords, while in WoS, the search was conducted based on the topic (TS). The search was further refined by language (English) and document type (article). No time restrictions were applied to ensure the inclusion of all relevant articles and to capture the field’s evolution from its initial stage. The search string retrieved a total of 841 articles, with 601 from Scopus and 240 from WoS.

### 2.2. Inclusion and Exclusion Criteria

Only empirical, peer-reviewed articles written in English, involving samples of HCPs (e.g., doctors, nurses) and focusing on telemedicine as a research topic, published up to 2023, were included in the analysis. Conceptual papers, conference proceedings, dissertations, protocols, review articles, and studies not focused on HCPs or telemedicine were excluded due to their limited contribution to the research questions and conceptual mapping. Similarly, studies addressing digital training/interventions for workers outside the healthcare context, or those mentioning telemedicine only incidentally, were also excluded. No restrictions were applied concerning the journal’s impact factor or the country of origin of the studies, to ensure a broad and inclusive overview of the empirical literature available in the field.

### 2.3. Screening Process

**Figure 1:**
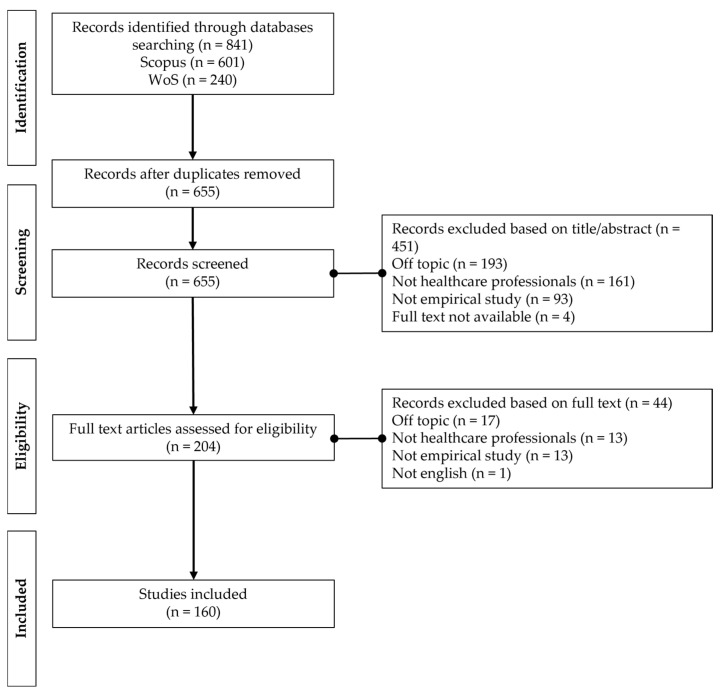
Flow diagram of records identified, screened, and included in the bibliometric analysis, according to PRISMA guidelines.

Following the completion of the semi-automated filtering process using database tools, a total of 841 articles were identified and subsequently checked for duplicates. After this step, 655 unique articles remained for further evaluation based on the established inclusion and exclusion criteria. In line with the Preferred Reporting Items for Systematic Reviews and Meta-Analyses (PRISMA) [30], two independent reviewers screened the titles and abstracts to assess their relevance to the research objectives. In cases of discrepancies between the reviewers, group discussions and detailed re-examinations of the studies were conducted until consensus was reached. Only articles unanimously deemed relevant were retained, leading to a refined dataset of 204 documents. A similar strategy was adopted during the final screening phase, in which the full text of the selected articles was reviewed to ensure compliance with the eligibility criteria [25]. As a result, 44 additional articles were excluded, leaving a final dataset of 160 articles. The screening process is illustrated in Figure 1.

### 2.4. Data Analysis

A bibliometric analysis was conducted using the Bibliometrix R-package (version 4.1.4, available at https://www.bibliometrix.org/home/index.php/layout/biblioshiny, accessed on 12 October 2024), a free and open source software that provides tools for quantitative bibliometric research [25]. Metadata (e.g., authors, citations, keywords) from the 160 selected articles were exported as a plain text file for subsequent analysis.

This study includes a performance analysis aimed at examining the characteristics of the sample and evaluating the influence of scientific output, including metrics such as the number of publications, citation rates, and the contributions of the different countries. These metrics are commonly used in bibliometric research to assess scientific productivity (e.g., number of publications), impact (e.g., citations), and the geographical and institutional distribution of knowledge. Their inclusion allows for a comprehensive overview of the research field [23]. Additionally, the most frequently used terms related to HCPs’ perspectives on telemedicine were analyzed. This was followed by co-word and thematic analyses to explore and map the intellectual structure of the research domain [31]. Specifically, co-word analysis assumes that the co-occurrence of keywords reflects the underlying themes within documents, thus allowing for the detection and visual representation of topic clusters in the field [26]. Thematic analysis was then applied to identify the main research themes and measure their relevance in relation to the development of the overall field [32].

## 3. Results

### 3.1. Data Description

This bibliometric analysis included a total of 160 articles published between 2001 and 2023. These articles were distributed across 119 different sources. In total, the analysis identified 483 unique authors from 43 different countries (Table 1).

### 3.2. Global Publication Trends

**Figure 2:**
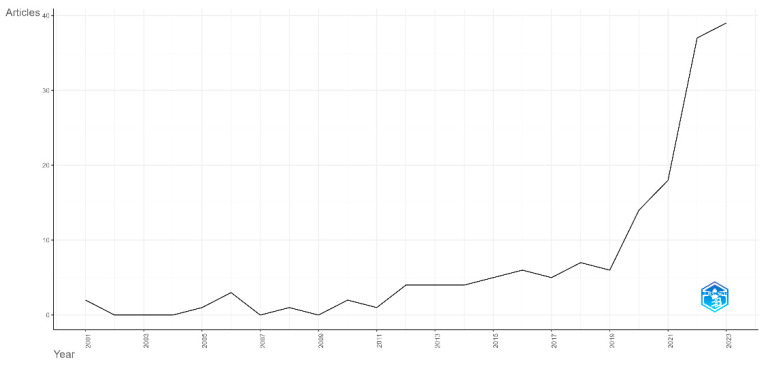
Annual scientific production.

The annual scientific production exhibited notable fluctuations in the number of published works. Initially, the field showed modest activity, with only 2 publications in 2001. However, several years passed without significant output, indicating that interest in telemedicine, especially concerning the point of view of HCPs, was in its early stages. From 2012 onwards, there was a steady increase in publications, peaking in 2020. Specifically, the number of publications rose from fewer than 10 per year between 2001 and 2019, to more than 14 per year starting in 2020. The upward trend continued, with 37 articles published in 2023, suggesting growing recognition of the importance of considering HCPs’ perspectives on telemedicine adoption. Figure 2 illustrates the publication trend.

### 3.3. Most Relevant Journals, Articles, Countries and Affiliations

An analysis of the sources revealed a small subset of journals that dominate the literature in this field (Table 2). Notably, journals such as the Journal of Medical Internet Research and BMC Health Services Research were identified as leading platforms for disseminating research on telemedicine from the HCPs’ perspective. The main disciplinary focus of each journal was identified using Scopus classification. Interestingly, all journals were in the health or medical domain, with no journals in psychology or humanities.

Table 3 presents the ten most cited articles. The most frequently cited article was published by Hennemann and colleagues [33], followed by the one written by Katzman and colleagues [34] and Villani and colleagues [35].

**Figure 3:**
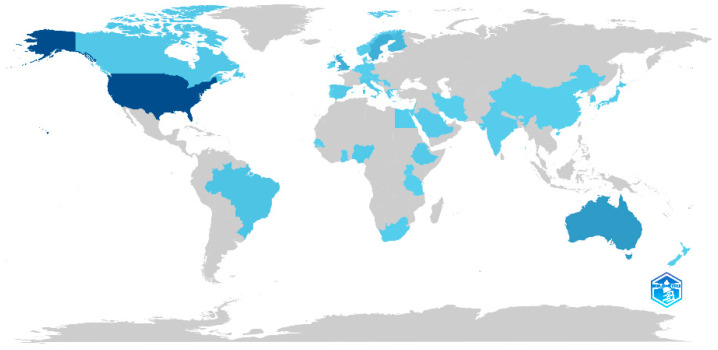
Scientific production by country. Different shades of blue indicate different productivity rates: dark blue = high productivity; grey = no articles.

The geographical distribution of research output indicates the leading role of certain countries in advancing telemedicine research from the perspective of HCPs (Figure 3). The United States emerged as the most prolific contributor, with the highest number of publications (n = 138). Other major contributors included Australia (n = 54), United Kingdom (n = 34), and European countries such as Sweden (n = 33) and Finland (n = 23).

**Figure 4:**
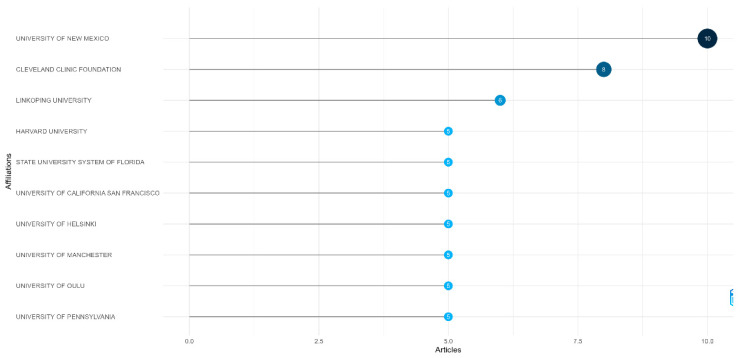
Most relevant affiliations. Different shades of blue represent varying productivity levels: dark blue indicates high productivity, while light blue indicates low productivity.

Focusing on affiliations, the University of New Mexico (n = 10) and the Cleveland Clinic Foundation (n = 8), both based in the United States, emerged as the most productive institutions. Ranked third was Linköping University (n = 6) in Sweden, where e-health is one of the key areas of research activity (Figure 4).

### 3.4. Most Relevant Words and Frequency over Time

**Figure 5:**
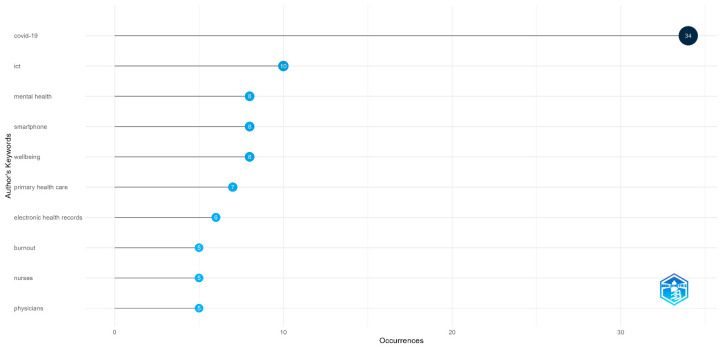
Words frequency. Different shades of blue represent varying frequency levels: dark blue indicates high frequency, while light blue indicates low frequency.

An analysis of authors’ keywords offered valuable insights into the dominant terms in the scientific literature. Notably, terms such as “COVID-19” and “ICT” were among the most frequently occurring words, underscoring their centrality to the discourse. Other frequent keywords included “mental health”, “smartphone”, and “wellbeing” (Figure 5).

### 3.5. Co-Word Analysis

A co-word analysis was conducted to identify the cognitive structure and conceptual linkages within the selected research domain [43]. To simplify the analysis, keywords related to the overarching term “telemedicine” were removed from the search query. Additionally, broad terms (e.g., factors) and those referring to research methodologies (e.g., qualitative research, cross-sectional studies) were excluded. Keyword spelling was standardized (e.g., singular/plural forms, hyphenation). Using the author’s keywords as the unit of analysis, this approach identifies terms (nodes) that frequently co-occur in literature and assesses their relationships. The size of each node reflects the keyword occurrence (i.e., betweenness degree; B), while the thickness of the connecting lines indicates the strength of co-occurrence (i.e., centrality degree; C). A total of 49 nodes and 9 clusters were identified.

**Figure 6:**
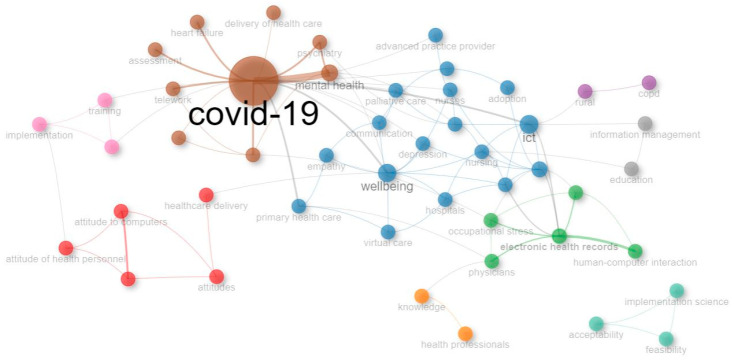
Co-word analysis.

Figure 6 shows that the “COVID-19” cluster emerged as the most relevant area (B = 510.02 and C = 0.01), with a strong connection with “Mental health”. This cluster also includes terms such as “Burnout” and “Psychiatry”. The “Wellbeing” cluster was the second most relevant (B = 217.84 and C = 0.01), showing several associations with the “COVID-19” cluster. It includes terms such as “ICT” (Information and Communication Technology), and “Primary health care”. The “Healthcare delivery” cluster appeared as the third most relevant (B = 96.36 and C = 0.01), with links to the “Wellbeing” cluster. This cluster includes terms such as attitudes and attitudes of health personnel. Notably, the “Acceptability” cluster (B = 0 and C = 0.5), which includes terms such as “Feasibility” and “Implementation science”, appeared disconnected from the other clusters.

### 3.6. Thematic Analysis

Using the approach outlined by Cobo and colleagues [32], a thematic map was created to highlight the key themes. The themes are positioned across four quadrants, defined by two factors: density and centrality. Density reflects the internal cohesion of a network, indicating how well-developed a theme is. Centrality, on the other hand, measures the extent to which a theme interacts with other themes, representing its importance in the overall development of the research area [32]. The intersection of the density and centrality axes produces four distinct quadrants, including different themes (i.e., motor themes, niche themes, emerging or declining themes and basic themes). In the thematic map, the size of each bubble reflects the frequency of word occurrences within each cluster, with larger bubbles representing more frequent themes [44].

**Figure 7:**
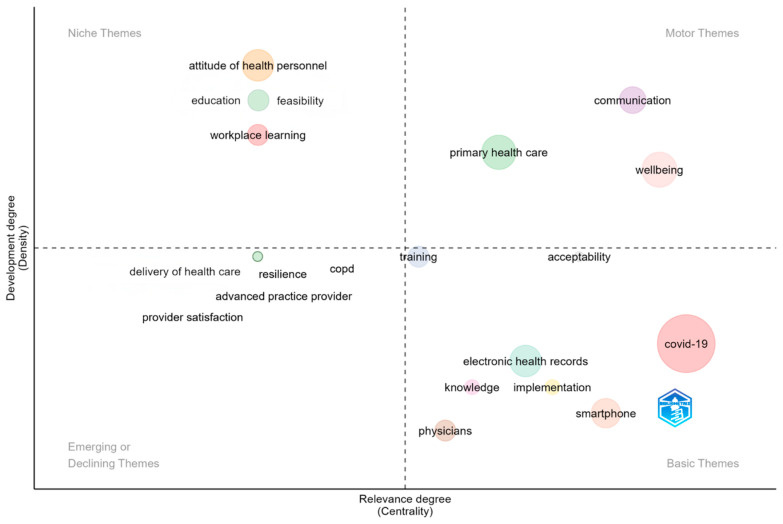
Thematic map.

Author’s keywords were used as the unit of analysis, and the following parameters were configured: number of words set to 300, minimum frequency set to 2, and number of labels set to 1. A total of 22 clusters emerged (Figure 7).

The first quadrant includes “motor themes”, characterized by high centrality and high density. These are well-established and crucial themes that shape the research field. Three motor themes were identified: “wellbeing”, “primary healthcare”, and “communication”, with the first two having similar bubble sizes. The “wellbeing” cluster refers to research on the impact of telemedicine on the wellbeing of both HCPs’ and patients. Studies have explored how telemedicine can affect HCPs’ wellbeing [45], as well as how online mental health programs can support nurses’ wellbeing [35,40]. Other research has investigated HCPs’ perceptions of how ICT use may improve or hinder patients’ wellbeing [46,47]. The “primary health care” cluster explores how telemedicine influences physicians’ work in primary care. For instance, Fernemark and colleagues [48] conducted an in-depth analysis of job demands, job control, and social support for physicians using digital consultations. Additionally, Björndell and Premberg [49] demonstrated that video consultations can enhance physicians’ work situations and wellbeing. Finally, “communication” emerged as a key theme, highlighting the importance of effective communication in telemedicine. Providing virtual care is challenging for physicians, who must reinforce their communication skills and their ability to manage misunderstandings [50,51]. Effective communication is also crucial for maintaining patient engagement [52].

The second quadrant highlights “niche themes”, characterized by both low density and low centrality, suggesting that these themes are either underdeveloped or becoming less relevant over time. The largest bubble within this quadrant is “Attitude of health personnel”, indicating that this is the most discussed area. This cluster focuses on how HCPs’ attitudes toward telemedicine can influence its implementation. Specifically, studies have identified key factors that affect the acceptance and perceived usefulness of various telemedicine approaches [37,42]. Similarly, the “feasibility” cluster includes studies examining HCPs’ opinions on the feasibility of different telemedicine methods [53,54]. The “education” cluster addresses the need for HCPs operating in digital environments to possess the knowledge, skills, and resources to provide optimal patient care through telemedicine. This cluster includes literature on identifying educational needs [55] and evaluating the effectiveness of telemedicine training programs [56]. The “workplace learning” cluster focuses on providing HCPs with training and interprofessional education programs to develop their skills. Closely related to telemedicine, workplace learning equips HCPs with the competencies needed to effectively use telemedicine tools, such as video consultations [57], while also fostering collaboration among professionals via telehealth [58].

The third quadrant includes “emerging or declining themes” which are underdeveloped and marginal. The “resilience” cluster—represented by a small bubble—encompasses research where digital health platforms were used to implement programs aimed at improving the resilience (i.e., a personal resource that supports individuals in managing stress and recover from adversities [59]) and wellbeing of HCPs [60,61]. This highlights that telemedicine services benefit not only patients but also HCPs, as digital platforms are used to improve their resilience and wellbeing. Moreover, “provider satisfaction” cluster includes articles aimed at investigating HCPs’ satisfaction with telemedicine and virtual care delivery [62,63].

The fourth quadrant features “basic themes”, which are characterized by high centrality and low density. These themes are central to the field but remain underdeveloped. The “COVID-19” cluster is represented by the largest bubble, signifying that it is the most prevalent theme in this quadrant. This cluster includes studies examining HCPs’ experiences with the adoption of telemedicine services during the pandemic [64,65]. The “physicians” cluster focuses on this specific group of HCPs. Several studies have explored in depth the experiences of physicians with digitalization and its impact on their work environment [41,66]. Additionally, the “knowledge” cluster underscores the importance of assessing physicians’ understanding of telemedicine services [67,68]. The “training” cluster focuses on academic research on interventions or programs designed to enhance HCPs’ skills in using telemedicine. Articles in this cluster explore the effectiveness of training aimed at improving communication skills among nurses in telehealth [69] and enhancing the virtual clinical skills of clinicians [70]. The presence of the “acceptability” cluster highlights the importance of evaluating HCPs’ willingness to use telemedicine and the variables that influence this predisposition. Studies in this cluster generally reveal high levels of acceptability, although specific concerns depend on the type of service. For instance, healthcare providers deemed telemedicine for medical abortion highly acceptable but expressed concerns about its safety and effectiveness [54]. In addition to users’ acceptability, the “implementation” cluster emphasizes the need to consider organizational and contextual factors that may affect the adoption of telemedicine services, such as telehealth for abortion care [71] or video consultations [57].

## 4. Discussion

The present bibliometric analysis aimed to provide a comprehensive overview of the current state of research on the implications of telemedicine for HCPs. While previous reviews on the adoption of telemedicine among HCPs exist [19,20], to the best of our knowledge, this is the first study to apply a quantitative, bibliometric approach to this specific area of research. By employing this method, we were able to uncover key trends and thematic clusters that qualitative analyses might not fully capture, thus offering a more comprehensive understanding of the research landscape.

Our analysis revealed a marked increase in scientific publications beginning in 2012, likely fueled by technological advancements such as the widespread adoption of telemedicine platforms and improvements in digital infrastructure. However, the most significant peak in publications occurred from 2020 onward, underscoring the profound impact of the COVID-19 pandemic. This theme emerged consistently across various analyses within this bibliometric study, emphasizing the pivotal role of COVID-19. During this period, telemedicine emerged as a key tool for healthcare delivery amidst lockdowns and physical distancing measures [6,72,73]. Notably, the volume of articles published in 2023 remains substantial, indicating that telemedicine’s relevance extends beyond the crisis and suggesting a shift toward more sustained and integrated adoption of these tools in HCPs’ work. Interestingly, other bibliometric analyses focused on telemedicine—such as those examining patient’s role [74] or specific healthcare areas [75]—have found similar surges in research output during the same period. This convergence across different aspects of telemedicine research points to a broader academic recognition of the field’s relevance.

The predominance of health and medical journals, such as the Journal of Medical Internet Research and BMC Health Services Research, suggests that research on the implications of telemedicine for HCPs is predominantly housed within the clinical sector. Notably, contributions from psychology or the social sciences are scarce, representing a gap in the literature that could provide valuable insights into the psychological, social, and organizational dimensions of telemedicine adoption and use.

Geographically, our analysis aligns with previous studies showing that research in the telemedicine field is primarily concentrated in high-income countries [74,76]. The United States, Australia, and the United Kingdom emerge as key contributors, followed by some European countries. This prominence likely reflects the advanced healthcare systems and robust technological infrastructures in these nations, which facilitate the adoption of telemedicine [77]. These countries also tend to have innovation-driven cultures that prioritize the rapid diffusion of new technologies [76,78]. Notably, our analysis also identified contributions from non-Western countries, including Nigeria, Ethiopia, India, and Japan. Although these countries currently have lower publication volumes, their presence in the dataset highlights a growing global interest in the implications of telemedicine for HCPs.

The co-word analysis revealed that “COVID-19” and “wellbeing” are frequently studied together, indicating that the recent literature has largely centered on how telemedicine implementation during the pandemic influenced HCPs’ wellbeing [64,65]. In contrast, there has been limited research examining “wellbeing” and “resilience”, highlighting an underdeveloped area that merits further investigation, particularly in high-stress environments such as emergency care. Furthermore, thematic analysis identified “wellbeing” as a motor theme, revealing a strong connection with mental health concerns such as burnout and stress, especially during the COVID-19 pandemic. Some studies explored the potential of digital interventions in supporting professionals’ psychological wellbeing—for instance, mobile-based mental health support or resilience training programs [35,40,60,61]. However, this theme remains underexplored, especially in relation to longitudinal effects or contextual moderators such as workload or organizational support. The analysis highlights a gap in understanding how telemedicine-induced stress might impact not only job satisfaction, but also quality of care, reinforcing the need for targeted strategies to protect HCPs’ mental health in digital environments.

**Figure 8:**
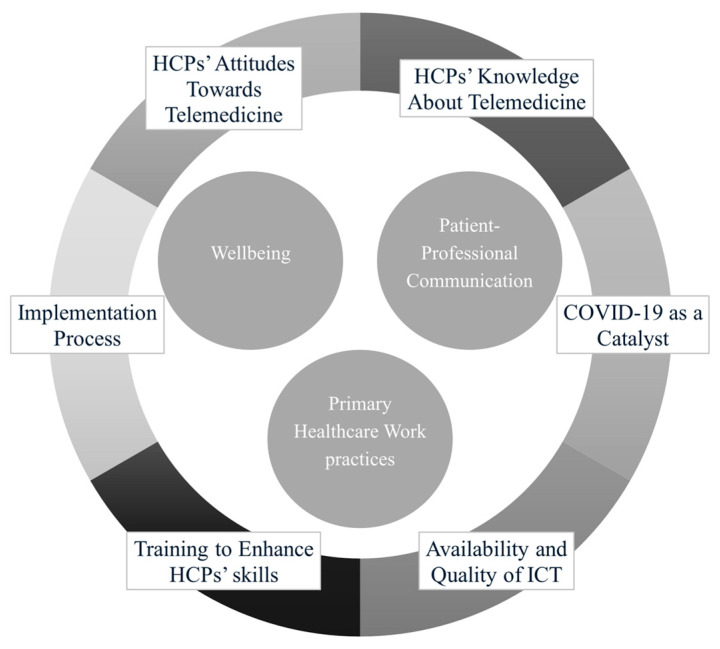
Integrative framework.

The co-word and thematic analyses allowed for the development of a framework (Figure 8) that provides an integrative structure summarizing the main trends in the literature. This framework synthesizes the key themes into an organized structure, offering a comprehensive view of the field. It highlights underexplored areas such as the relationship between resilience and wellbeing and provides insights into future research and implementation strategies, helping to identify key factors that can improve HCPs’ experiences and the effectiveness of healthcare delivery.

The intellectual structure of the field reveals three core themes: wellbeing, primary healthcare work practices, and patient–professional communication. These findings suggest that the wellbeing of HCPs is a central dimension in the study of telemedicine adoption. For instance, Werkmeister and colleagues [45] highlighted how constant connectivity and the blurring of boundaries between work and private life can deplete HCPs’ psychological resources. Additional evidence suggests that the digitization of healthcare can lead to increased job demands, such as time pressure and cognitive overload associated with poor usability of digital platforms [12,38]. Similarly, Fernemark and colleagues [48] found that virtual care models can alter the balance between job demands, control, and support, with potential consequences for HCPs’ wellbeing. Specific features of telemedicine may introduce new stressors that heighten the risk of burnout among HCPs [16]. These include not only the increased administrative burden and patients’ difficulties in using the technology [7], but also the cognitive strain associated with managing complex virtual interactions. Notably, while telemedicine may reduce some administrative tasks, it can simultaneously intensify mental workload by requiring the continuous adaptation of work practices and communication strategies in a digital context.

Thus, in addition to its impact on wellbeing, the adoption of telemedicine has significantly transformed daily clinical practice, particularly in primary care, offering both opportunities and challenges. For instance, Björndell and Premberg [49] demonstrated that video consultations can enhance physicians’ work situations by offering greater flexibility in managing patient appointments and reducing travel time, especially in rural areas. However, the shift to telemedicine can also lead to feelings of isolation from colleagues and reduce opportunities for in-person collaboration, which is often a key source of professional support in primary care settings. Similarly, communication between patients and HCPs in telemedicine settings presents significant challenges [50,51]. Ensuring effective communication is essential for improving the quality of telemedicine care, particularly in promoting patient engagement and enabling successful digital interactions [52]. This aligns with previous research highlighting the difficulties of web-based patient–professional communication [79] and reinforces the pivotal role of strong patient–professional communication in supporting medication adherence and achieving positive clinical outcomes [80].

Both contextual and personal factors shape these core themes and their interactions. Among the contextual factors, the emergence of “COVID-19” as a basic theme reflects the pandemic’s role as a catalyst for telemedicine adoption, with a significant impact on HCPs’ wellbeing. The pandemic intensified these issues, as HCPs were required to rapidly adapt to remote care settings, often without sufficient support or training [21]. The success of telemedicine hinges on the quality and availability of “ICT” infrastructure (including smartphones), which significantly affects both HCPs’ experiences and patient outcomes. Furthermore, organizational policies and practices, such as providing HCPs with adequate “training” to develop both technical and soft skills, are essential for promoting the successful “implementation” of telemedicine. Additionally, training can help improve HCPs’ knowledge and awareness of telemedicine, enabling them to better cope with the changes associated with its introduction.

Among personal factors, HCPs’ “attitudes toward telemedicine” are crucial in determining its success and influencing their wellbeing. In addition to attitudes, the level of “knowledge” that HCPs possess about telemedicine services shapes their experiences and perceptions of care quality. A positive attitude and greater familiarity with telemedicine can foster successful adoption, while higher technical proficiency increases the likelihood that HCPs will advocate for improvements in ICT and training within their organizations.

These findings suggest that telemedicine is a double-edged sword. On the one hand, its flexibility and enhanced access to care have been celebrated as key benefits, especially during the pandemic. On the other hand, our findings confirm that these advantages are accompanied by significant trade-offs that may influence HCPs’ wellbeing [48,81].

Existing models used to study telemedicine from the perspective of HCPs, such as the Technology Acceptance Model [82] and the Unified Theory of Acceptance and Use of Technology [83], primarily focus on individual cognitive factors (e.g., perceived usefulness, ease of use, or behavioral intention) influencing telemedicine acceptance and the intention to use it [14,22,84]. While these frameworks provide valuable insights into the adoption process, they overlook the broader impact of telemedicine on HCPs’ wellbeing and daily clinical practice.

By contrast, the framework proposed in this study emphasizes wellbeing as a central dimension in the telemedicine experience. It integrates contextual (e.g., pandemic-related changes, infrastructure, organizational support) and personal factors (e.g., attitudes, knowledge), highlighting the complex interplay between technology use and professional sustainability. This broader perspective advances the field by reframing telemedicine as a complex organizational transformation rather than a mere technological intervention.

The main takeaway from this framework is that successful telemedicine implementation requires more than just technological readiness. The wellbeing of HCPs is not a side effect of telemedicine implementation, but a pivotal outcome that directly impacts sustainability and quality of care. Promoting HCPs’ mental health should therefore be considered a strategic priority in any telemedicine integration program. In addition, resistance to change remains a significant challenge when introducing telemedicine services. This resistance can stem from various factors, such as uncertainty about new technologies, fear of increased workload, or concerns about diminished patient relationships [7,85]. Therefore, policymakers and healthcare leaders should foster a culture of adaptability, providing adequate training, and promoting the value of telemedicine to both professionals and patients. By integrating both technological and interpersonal aspects of telemedicine, they can ensure that the benefits of digital healthcare are maximized while minimizing its potential drawbacks for professionals.

### Implications for Practice

While training in digital skills is essential, our findings suggest that technical preparation alone is not sufficient to support HCPs in the transition to telemedicine. Specifically, issues such as HCPs psychological wellbeing and digital communication skills emerged as key themes in the literature.

Several studies highlight the need for more holistic and multidimensional support strategies. For instance, some institutions have implemented digital mentorship programs and peer-support groups to foster mutual learning and reduce the sense of isolation often experienced in remote work environments [57]. Other promising practices include micro-training sessions focused on communication skills in virtual settings—such as teach-back techniques or emotional attunement in video consultations [69]—which can mitigate misunderstandings and enhance patient engagement. Therefore, healthcare leaders should invest in structured training programs that not only enhance digital competencies but also address concerns related to work overload, job satisfaction, and role clarity. In line with our thematic findings, the promotion of a supportive organizational culture—one that values flexibility, professional autonomy, and ongoing feedback—is essential in reducing resistance and increasing acceptance of digital tools.

Moreover, initiatives aimed at improving telemedicine adoption should be co-designed with healthcare staff, acknowledging their perspectives and fostering a sense of ownership. Clear communication about the purpose, benefits, and expected changes related to telemedicine use can also help mitigate perceived threats, such as the loss of relational quality with patients or increased administrative burden.

In addition to skill-based training, healthcare organizations should also invest in psycho-educational interventions that allow professionals to process the deeper shifts in their professional identity, role boundaries, and interpersonal dynamics brought about by telemedicine. Creating spaces for structured reflection (e.g., interprofessional debriefing sessions or facilitated workshops) can provide emotional relief and strengthen professionals’ capacity to adapt constructively to digital change. These supportive interventions are particularly relevant in high-stress environments, where the adoption of telemedicine may otherwise exacerbate existing psychological burdens.

By integrating these organizational and relational aspects with technological innovation, healthcare institutions and policymakers can ensure that telemedicine becomes a sustainable and accepted part of clinical practice, rather than a source of additional strain or uncertainty for professionals.

## 5. Conclusions

This bibliometric analysis offers a comprehensive overview of the research landscape on the implications of telemedicine adoption among HCPs. The findings indicate a steady increase in scientific output starting in 2012, with a notable surge in publications from 2020 onward, reflecting the impact of the COVID-19 pandemic as a catalyst for telemedicine implementation. The findings of this bibliometric analysis enabled the development of a conceptual framework that synthesizes the core themes of the field. While telemedicine provides numerous advantages, such as improved access to care and increased flexibility, it also poses challenges for wellbeing, primary healthcare work practices, and communication between patients and professionals. Contextual and personal factors significantly influence how HCPs experience telemedicine. In summary, this study contributes to the literature by revealing that the wellbeing of HCPs is a core issue in telemedicine implementation—often neglected in prior research. Future strategies must balance technological benefits with the psychological needs of professionals to ensure effective and humane digital healthcare delivery. By uncovering key conceptual insights into the intellectual structure of this research field, this bibliometric analysis highlights not only well-established themes but also underexplored areas, providing a foundation for future research directions.

## 6. Suggestions for Future Research

The findings of this bibliometric analysis identify key areas for future research on the implications of telemedicine for HCPs. The analysis of the main journals suggests the need for a more heterogeneous and multidisciplinary approach, incorporating perspectives from psychology and social sciences, which have been underrepresented in the current literature. Our findings underscore the close connection between telemedicine adoption and HCPs’ wellbeing and work outcomes. Future research should delve deeper into this area, particularly through longitudinal studies that examine the long-term impact of telemedicine on HCPs, including how the use of these technologies influences their work practices, stress levels, and overall wellbeing.

Additionally, qualitative studies exploring how professional identity is reshaped in telemedicine contexts could provide valuable insights into the evolving roles and relationships between HCPs and patients. The shift from in-person to virtual care may alter professionals’ perceptions of their roles and responsibilities [4]. Understanding these changes is essential to support HCPs in maintaining their professional identity in a rapidly digitalizing environment.

Themes related to resilience and provider satisfaction remain underdeveloped in the current literature. Future studies should investigate how telemedicine can be utilized to support HCPs’ mental health and resilience, particularly in high-stress environments (e.g., first aid). Moreover, our analysis reveals that most research on telemedicine is concentrated in high-income countries, with limited representation from low-income countries. Future research should explore how telemedicine is implemented in resource-limited settings, where its impact on HCPs may differ significantly from that in high-income contexts.

Furthermore, this bibliometric analysis revealed a lack of research on the effects of telemedicine on collaboration within healthcare teams. Future studies should examine the dynamics of virtual teamwork, the development of collaborative skills in digital environments, and how telemedicine either facilitates or hinders effective interprofessional collaboration. As telemedicine becomes integrated into healthcare systems, it is crucial to explore the role of training in equipping HCPs with both the technical and soft skills necessary for successful telemedicine use. Future research should assess the effectiveness of training programs and identify best practices for skill development.

In addition, the role of organizational policies and support mechanisms in facilitating telemedicine adoption requires further exploration. Future research should explore ways in which organizations can effectively support HCPs through policies, training, and resources that improve telemedicine adoption, enhance job satisfaction, and reduce burnout. Organizational factors such as leadership, culture, and resource allocation should be examined in relation to the successful implementation of telemedicine.

Finally, while the present study provides a comprehensive overview of the intellectual and thematic landscape of the literature on telemedicine from the perspective of HCPs, it does not include a systematic classification of the reviewed studies in terms of epidemiological methods or study designs. Future research could build upon these findings by conducting a systematic review. Such an approach would offer deeper insights into the robustness of the existing evidence base and complement the bibliometric and content-driven perspective presented here.

## Figures and Tables

**Table 1:** Overview of the contributions.

	Dataset Features	
General information	Timespan	2001:2023
	Journals	119
	Documents	160
	Annual growth rate (%)	14.46
	Average citations per document	14.03
	Average citations per year per doc	3.83
	References	4895
Document contents	Author’s keywords	600
Authors	Authors	905

**Table 2:** Most relevant journals.

	n	Highest Percentile (Source: Scopus)
Journal of Medical Internet Research	10	Health Informatics
BMC Health Services Research	7	Health Policy
International Journal of Environmental Research And Public Health	6	/
BMC Medical Informatics And Decision Making	4	/
BMJ Open	3	General Medicine
Plos One	3	Multidisciplinary
Telemedicine Reports	3	Medicine (miscellaneous)
Australian Journal of Primary Health	2	/
Frontiers in Digital Health	2	/
Health Informatics Journal	2	Health Informatics

N = number of published articles.

**Table 3:** Top manuscript by citation.

Ranking	Article	Number of Citations	Number of Citations per Year
1	Hennemann et al. [33]	123	15.38
2	Katzman et al. [34]	94	8.55
3	Villani et al. [35]	85	7.08
4	Maguire et al. [36]	81	8.1
5	Moeckli et al. [37]	71	1.39
6	Vehko et al. [38]	67	11.16
7	Srinivasan et al. [39]	62	12.4
8	Bolier et al. [40]	61	5.54
9	Grünloh et al. [41]	55	6.11
10	Ruiz Morilla et al. [42]	54	6.75

## Data Availability

The data associated with this research may be available upon reasonable request.
